# Consideration of specific key points improves outcome of decompression treatment in patients with endocrine orbitopathy: pre-/post-OP comparison and biomechanical simulation

**DOI:** 10.1186/s40001-022-00709-9

**Published:** 2022-06-13

**Authors:** Matthias Krause, Michael-Tobias Neuhaus, Ina Sterker, Alexander K. Bartella, Annika Schönfeld, Bernd Lethaus, Rüdiger Zimmerer, Evgeny Gladilin

**Affiliations:** 1 Department of Oral and Maxillofacial Surgery, Leipzig University, Liebigstraße 12, 04103 Leipzig, Germany; 2 Department of Ophthalmology, Leipzig University, Liebigstraße 12, 04103 Leipzig, Germany; 3 Applied Bioinformatics, German Cancer Research Center, Berliner Str. 41, 69120 Heidelberg, Germany; 4 Present Address: Leibniz Institute of Plant Genetics and Crop Plant Research, Corrensstrasse 3, OT Gatersleben, 06466 Seeland, Germany

**Keywords:** Endocrine orbitopathy, Decompression surgery, Computer tomography data, Biomechanical simulation

## Abstract

Endocrine orbitopathy is typically treated by resecting orbital walls. This procedure reduces intraorbital pressure by releasing intraorbital tissue, effectively alleviating the symptoms. However, selection of an appropriate surgical plan for treatment of endocrine orbitopathy requires careful consideration because predicting the effects of one-, two-, or three-wall resections on the release of orbital tissues is difficult. Here, based on our experience, we describe two specific orbital sites (’key points’) that may significantly improve decompression results. Methodological framework of this work is mainly based on comparative analysis pre- and post-surgery tomographic images as well as image- and physics-based simulation of soft tissue outcome using the finite element modelling of mechanical soft tissue behaviour. Thereby, the optimal set of unknown modelling parameters was obtained iteratively from the minimum difference between model predictions and post-surgery ground truth data. This report presents a pre-/post-surgery study indicating a crucial role of these particular key points in improving the post-surgery outcome of decompression treatment of endocrine orbitopathy which was also supported by 3D biomechanical simulation of alternative two-wall resection plans. In particular, our experimental results show a nearly linear relationship between the resection area and amount of tissue released in the extraorbital space. However, a disproportionately higher volume of orbital outflow could be achieved under consideration of the two special key points. Our study demonstrates the importance of considering natural biomechanical obstacles to improved outcomes in two-wall resection treatment of endocrine orbitopathy. Further investigations of alternative surgery scenarios and post-surgery data are required to generalize the insights of this feasibility study.

## Background

Endocrine orbitopathy (EO), also known as Graves’ orbitopathy (GO) or thyroid eye disease (TED), is the main extrathyroidal manifestation of Graves’ disease and is found in about 25% of patients at diagnosis often as a mild and self-remitting condition. Severe forms like dysthyroid optic neuropathy (DON) affects 3% to 5% of patients. It presents as an ocular disease, causing aesthetic disfigurement and functional deficits, and may lead to diplopia or even a loss of vision. The complex surgical treatment of endocrine orbitopathy (EO) remains an important option when thyroid surgery with/without radiation and other conservative approaches, such as corticosteroid therapy and immunosuppressive drugs, fail [1–3]. The first step, orbital decompressions surgery, results in an increase in orbital volume and is achieved by removing the orbital walls, thus reducing intraorbital pressure by releasing intraorbital tissue. This type of surgery can improve disfiguring symptoms such as exophthalmos, eyelid retraction and restriction of eye movements. However, decision-making during preoperative planning of EO surgery is challenging because the impact of one-, two-, or three-wall resection on the release of orbital tissues is difficult to predict. The choice of surgical technique depends on the surgeon’s preference and objective criteria, resulting in patient-specific surgical plans that consider the patient’s anatomy, orbital pathology, and general treatment goals.

Realistic assessment of the postoperative outcome of the surgical EO correction (with respect to reduction of protrusion and change in soft tissue) is the ultimate goal of surgical planning. However, selecting a surgical plan for orbital decompression is challenging because the effects of orbital wall resection on the release of orbital tissues are difficult to predict. Many variations of orbital decompression surgery have been introduced for the treatment of this condition. To date, thyroid orbital surgeons have developed specific and individual surgical procedures to best predict favourable surgical outcomes [4]. Several studies have investigated the effect of orbital bony decompression through the deep lateral wall [5], and removing the bony wall is performed first as a deep side wall and then as a middle and floor wall [6].

While resection of larger areas of orbital walls is certainly correlated with larger amount of release soft tissue, maximization of the overall therapeutic benefit with minimization of therapeutic morbidity should be the aim of the surgical plan [7]. In fact, the most rigorous three-wall resection approach to EO treatment is known to be associated with a higher complication rate, especially in the case of medial wall resection [8–11]. Consequently, here we focus on two-wall resection technique and report that consideration of two key anatomical points significantly improves the post-surgery outcome of two-wall resection in comparison to surgery scenarios that do not take them into consideration. These two key points are (i) the lacrimal keyhole at the lateral orbit (key point 1, kp1) and (ii) the basin of the inferior orbital fissure with the cranial part of the lateral sinus wall (key point 2, kp2) [12]. The evidence for importance of these two key points comes from comparative analysis of pre- vs. post-OP tomographic images as well as a 3D biomechanical simulation. Our hypothesis is that wide resection of the lateral and infraorbital orbital walls under consideration of the above key points can predictably achieve a good result of two-wall decompression avoiding additional re-decompression surgery.

Early approaches to computational modelling of orbital mechanics demonstrated feasibility of for anatomy- and physics-based simulations for quantitative analysis of biometric parameters orbital decompression surgery including correlations between the surface and volume of the resected orbital walls and the displacement of the bulbus [13, 14]. However, the early studies did not include postoperative data, and, thus, remained hypothetical. More recently computational frameworks for the simulation of individual surgical procedures in cranio-maxillofacial and orbital surgery were presented [15, 16]. Here, we use a 3D image and biomechanics-based simulation to investigate the two-wall resection plans, which occur in 72.5% of our orbital decompression cases [17], focusing on the impact of the two key points associated with improved postoperative outcomes.

This manuscript is structured as follows. First, we present and discuss the results of our comparative pre-/post-surgery and computational simulation studies. Then, the methodological framework of this work including study design, image processing and computational simulation of decompression surgery is described.

## Results

### Characterization of the study population

Table 1 gives a summary of the study population including four patients who underwent two-wall orbital decompression (lateral and floor walls) which had to be followed by additional re-decompression treatment because of insufficient results of the first two-wall decompression. Details on the study design and selection criteria can be found in the Methods section (study design).

**Table 1:** Demographic and biometric information of patients who underwent re-decompressions in the region of lateral orbital wall (key point 1) and lateral floor wall (key point 2) for treatment of endocrine orbitopathy

Pat.	Sex	Age	$\hbox {V}_r$	$\hbox {V}_r$	$\hbox {V}'_r$	$\hbox {V}'_l$	$\hbox {dV}_r$	$\hbox {dV}_l$	$\hbox {B}_r$	$\hbox {B}_l$	$\hbox {B}'_r$	$\hbox {B}'_l$	$\hbox {D}_r$	$\hbox {D}_l$
1	f	57	–	**47.7	–	**57.8	–	10.1		21	–	18	–	3
2	m	66	*36.7	–	*39.1	–	*3.1	–	16	–	10	–	6	–
3	f	55	*32.3	–	*35.9	–	*3.6	–	27	–	17	–	10	–
4	f	48	*34.0	–	**38.6	–	**4.6	–	28		22	–	6	–

V = pre-surgery orbital volume in cm$^3$; V’ = post-surgery volume of orbital tissue in cm$^3$; dV = V’-V; B and B’ = pre- and post-surgery bulbus distance (Hertel value) in cm, respectively; D = bulbus displacement (Hertel value) in cm; * key point 1; ** key point 2. r/l indicate right and left orbita

**Fig. 1:**
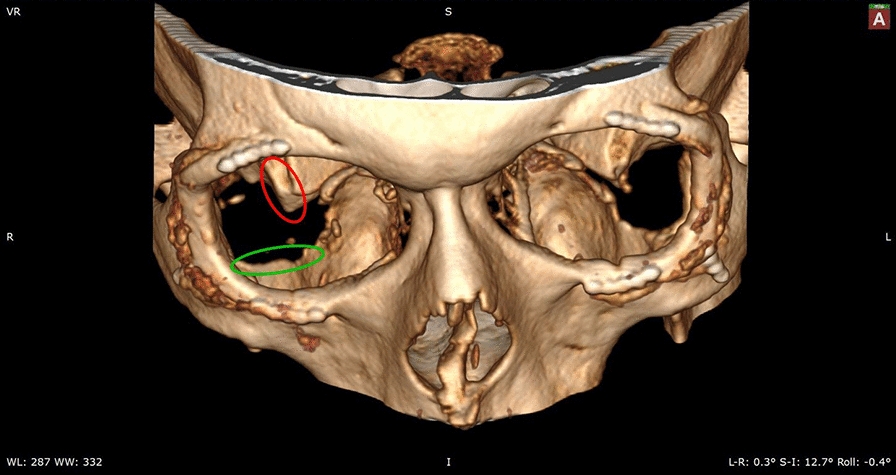
Visualization of post-orbital decompressions surgery CT. The post-orbital decompressions surgery CT (patient 2) shows insufficiently resected key point 1 on the right side (red circle) vs. well-resected key points on the left site. The green circle on the right side indicates the well resected key point 2

### Clinical observations

**Fig. 2:**
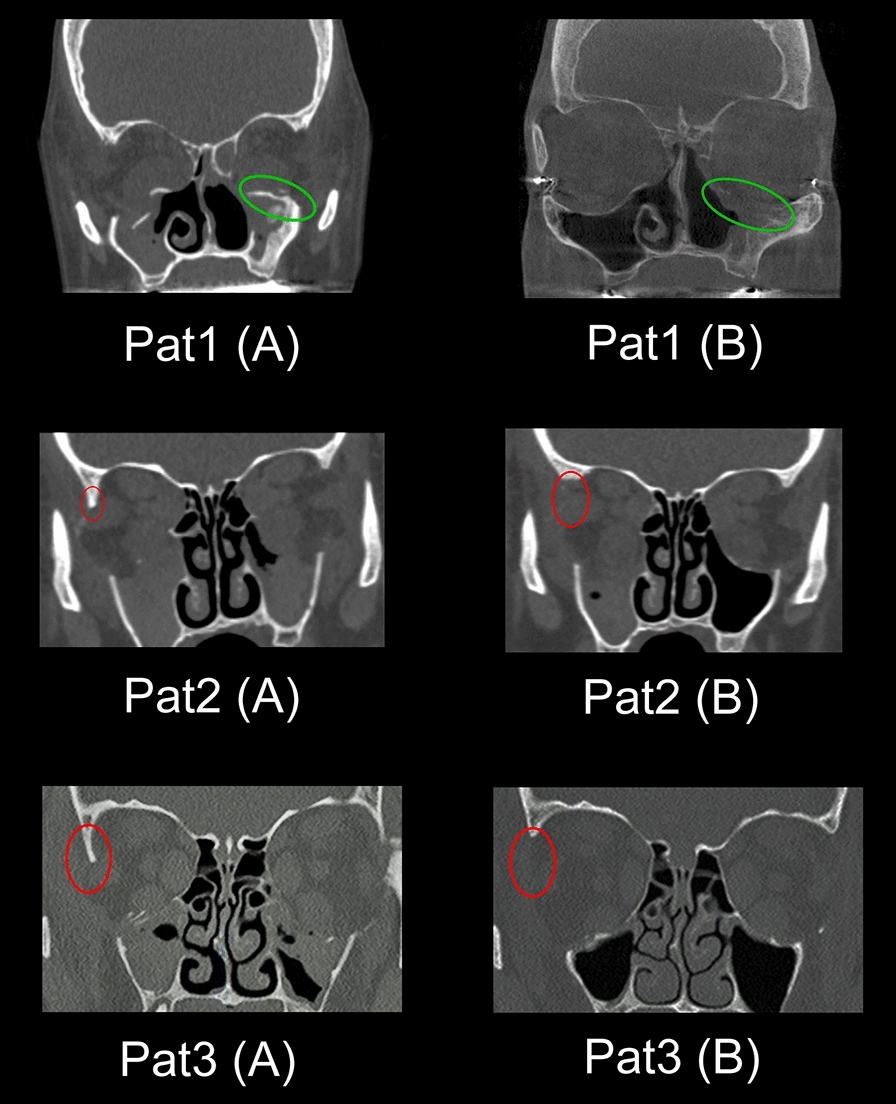
Post-surgery cross-section images. Cross-section images of post-orbital decompression (left **A** column) and re-decompression (right **B** column) CT data for three patients (no. 1, 2, 3 in Table 1. Green ellipses indicate remaining **A** re. resected **B** key point 2 in CT cross-section images of the patient 1. Red ellipses indicated remaining **A** re. resected **B** key point 2 in CT cross-section images of patients 2 and 3

It was observed that superior surgery outcome in all four patients could be achieved only after additional re-decompression treatment under consideration of two special key points in lateral and floor walls; see Fig. 1. In particular, postoperative computer tomography scans showed insufficient resection results of either the deep lateral orbital wall or lateral floor wall regions, which correlated with clinically inadequate reduction of the protrusions; see Fig. 2. All four patients who underwent orbital re-decompressions surgery under consideration of these two key points had satisfactory results with improvement of protrusion, reduction of orbital pressure sensation and achievement of a symmetrical orbital appearance.

### Biomechanical simulation of soft tissue outcome

**Fig. 3:**
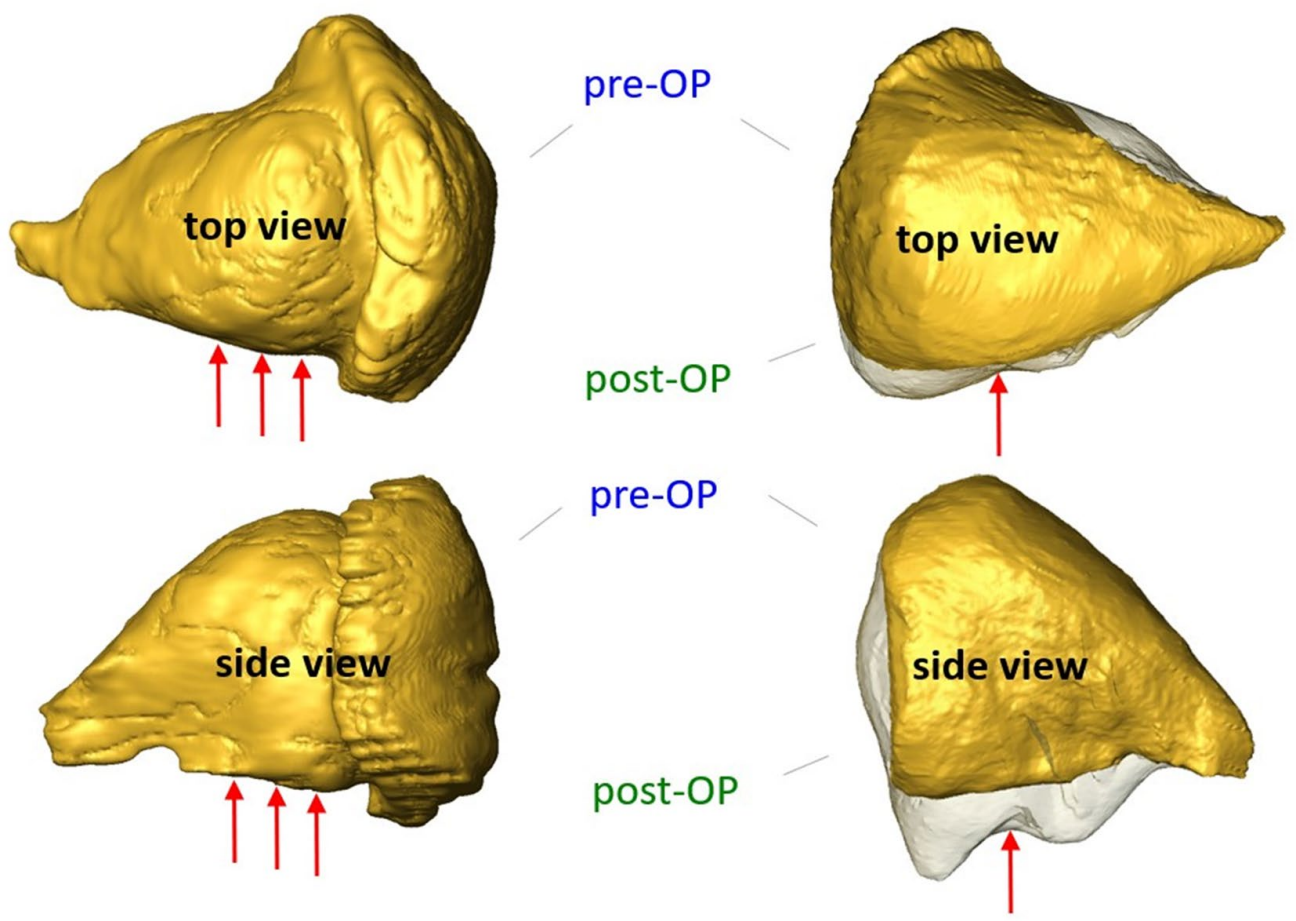
Reconstruction of boundaries of pre- vs. post-surgery orbital tissue. Surface models of pre- and post-surgery orbital tissue in top (top) and side (bottom) views. Red arrows indicate location of key points 1 (top view) and 2 (side view). The figures in the right column indicate the location of anomalies in outflow of orbital tissue (transparent surfaces) that are related to mechanical obstacles

To dissect the mechanistic cause of a disproportionately larger soft tissue outcome after consideration of two relatively small key points in lateral and floor walls, a biomechanical simulation of different scenarios of orbital wall resection was performed. For this purpose, pre- and post-surgery 3D CT datasets of a 67-year-old patient (no. 2 in Table 1) with EO and two orbital wall resections were selected. Figure 3 shows the pre- and post-surgery models of the patient’s orbital walls including positions of two key points.

**Fig. 4:**
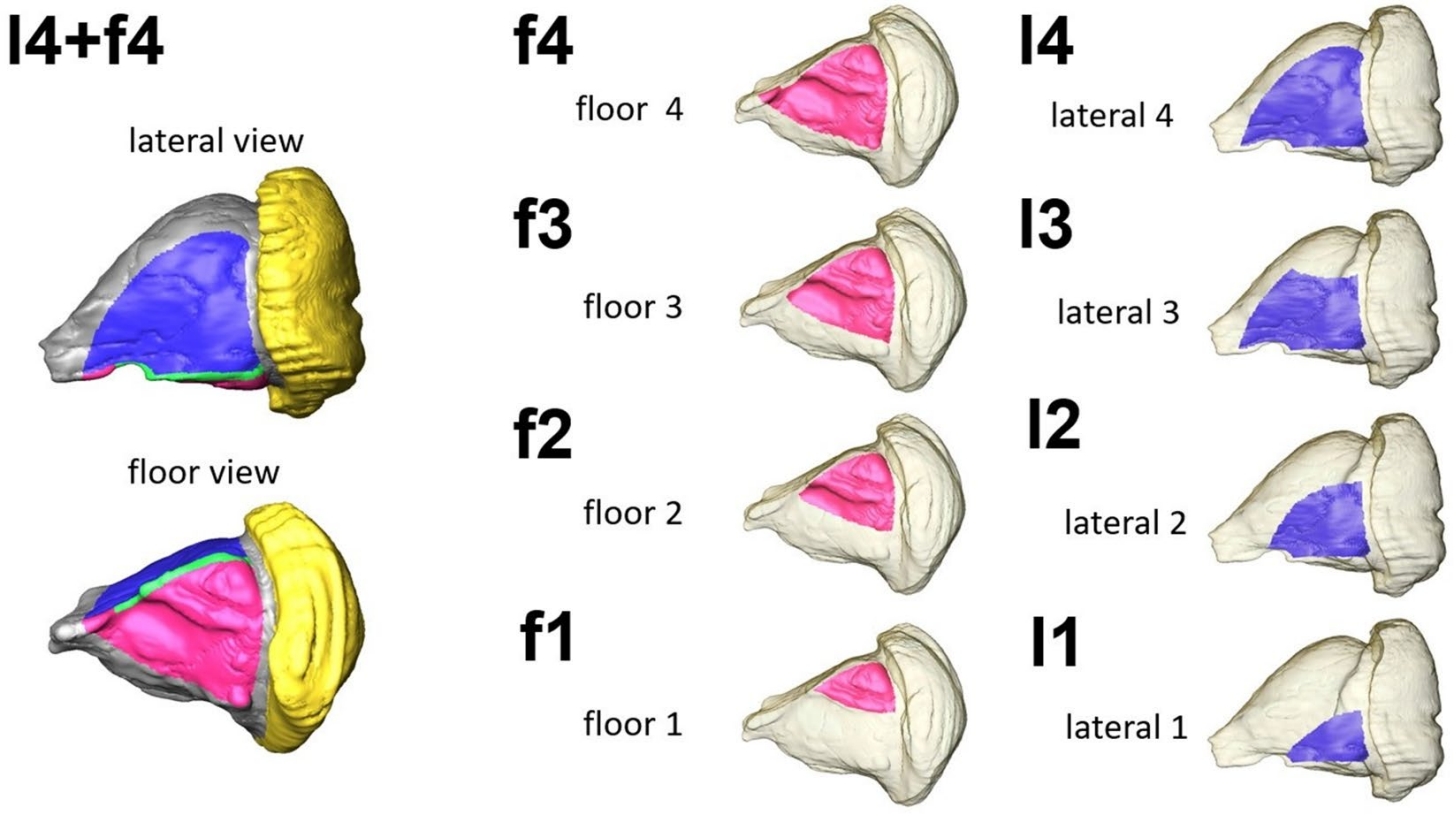
Visualization of simulated scenarios of graded decompression. Pre-surgery orbital model including segmented patches corresponding to lateral side (blue), floor (red), lateral key point 1 (yellow), and floor key point 2 (green) resection wall scenarios for comparative simulation of resection areas at the orbital floor (f1–f4) and lateral walls (l1–l4). Scenario l4+f4 shows the resection areas on the lateral and floor walls 4 with the key points

First, unknown material parameters and boundary conditions were estimated from the minimum difference between predicted and real post-surgery volume of release soft tissue as described in . This minimum was found for the following values of free model parameters: the relative muscle stiffness (i.e. the ratio between muscle and soft tissue stiffness) = 5, the effective Poisson ratio = 0.1, the ratio between the magnitude of forces acting on the bottom and lateral walls = 2.5. Higher stiffness of muscle tissue in comparison to fat tissue appears to be feasible. Also the fact that forces acting on the widely free bottom wall are effectively stronger than on the lateral wall which directly connected to lateral muscle and connective tissues layers comes not unexpected. The relatively low value of the Poisson ratio obtained from the simulation appears to contradict to typically higher range for low-compressible soft tissues (0.3-0.5). However, we treat it as an “effective macroscopic parameter” and not necessarily as compressibility of soft tissue. In particular, one should consider that several different physical and physiological mechanisms contribute to the difference between pre- and post-surgical CT images, which was used as a fitting criterion in our simulation. Important is the notion that the orbital space, which is computationally described as a closed domain with a preserved mass, is, in reality an open system which is exposed to additional flow/pressure of liquid from orbital blood vessel contributing to mechanical tissue expansion in the extraorbital space. All subsequent simulations were carried out using the above set of model parameters. Thereby, several different surgical scenarios were simulated, including four one-wall (lateral and floor), and two two-wall resections with particular attention given to key points. Figure 4 shows an overview of all the simulated decompression scenarios.

**Fig. 5:**
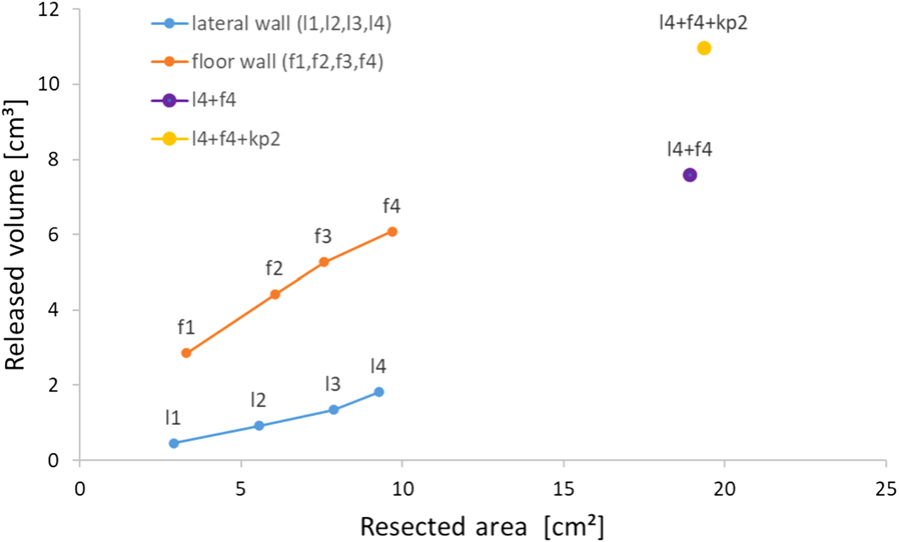
Plots the relationship between the released volume of orbital tissue vs. the area of resected walls. Summary of the computationally simulated relationship between the area of the resected orbital walls and the amount of released orbital tissue for the different one- and two-wall resection scenarios shown in Fig. 4. The points indicate the results obtained from computer simulations, whereas interpolating lines were introduced only for visualization (i.e. no continuous measurements between 1-4 points were performed)

Thereby, the amount of released soft tissue was computed for each of these scenarios and their combinations. These scenarios are as follows. Lateral wall 1 (l1) was a small resection area of 2.91 cm$^2$. This led to a simulated volume increase of 0.5 cm$^3$ (from 59.5 to 60.0 cm$^3$). The lateral wall 2 (l2) 5.5 cm$^2$ resection area simulated a volume increase from 59.5 to 60.5 cm$^3$ (1.0 cm$^3$). The 7.8 cm$^2$ lateral wall resection (l3) resulted in a simulated volume increase of 1.3 cm$^3$ (from 59.5 to 60.9 cm$^3$). The largest lateral area resected (l4) included kp1 and resulted in a simulated volume increase from 59.5 cm$^3$ to 61.4 cm$^3$ (1.8 cm$^3$), which corresponds to the relative volume increased by 26.2%. Similar results, but with larger areas and volumes, were revealed by the simulations of the floor walls. f1 had the smallest area (3.3 cm$^2$) and resulted in a simulated increase of volume by approximately 3 cm$^3$ (from 59.5 to 62.4 cm$^3$). f2, with a simulated area of 6.0 cm$^2$, increased the volume by 4.5 cm$^3$ (from 59.5 to 64.0 cm$^3$). Similar and approximately linear increases resulted from the simulation of areas f3 and f4; f3, with an area of 7.5 cm$^2$, simulated a volume increase of 5.2 cm$^3$ (from 59.5 to 64.3 cm$^3$), and f4, with an area of 9.6 cm$^2$, simulated a volume increase of 6.0 cm$^3$ (from 59.5 to 65.6 cm$^3$). f4 and l4 together resulted in an expected volume expansion of 7.5 cm$^3$ (from 59.5 to 67.1 cm$^3$). Expansion of kp2 resulted in a further volume expansion of 30.9% (11 cm$^3$, from 59.5 to 70.5 cm$^3$). Our simulation results show a nearly linear volume expansion for both simulated orbital surfaces laterally and in the floor area, with a stronger volume expansion in the floor area. However, when comparing these simulations to similar surgical scenarios without key point resections, a 26% larger volume expansion was observed with kp1 in the lateral wall, and a 30% larger volume expansion was observed with kp2 in the orbital floor. A summary of all simulation results including the relationship between the area of the resected walls and the amount of decompressed orbital tissue for all simulated scenarios can be found in Table 2 and Fig. 5.

**Table 2:** Summary of computational simulations of the orbital tissue release using the pre- and post-surgery models of patient 2 for the different one- and two-wall resection scenarios shown in Fig. 4

Resection	Scenario	Area	V pre	V sim	dV	dV rel
Lateral area	l1	2.91	59.60	60.06	0.46	25.21
Lateral area	l2	5.55	59.60	60.52	0.92	50.85
Lateral area	l3	7.87	59.60	60.94	1.34	73.84
Lateral area	l4=l3+kp1	9.28	59.60	61.41	1.81	100.00
Floor area	f1	3.31	59.60	62.46	2.86	46.99
Floor area	f2	6.04	59.60	64.01	4.41	72.49
Floor area	f3	7.58	59.60	64.87	5.27	86.58
Floor area	f4	9.69	59.60	65.68	6.08	100.00
L+F areas	l4+f4	18.92	59.60	67.18	7.58	69.13
L+F areas	l4+f4+kp2	19.36	59.60	70.56	10.97	100.00

V pre = pre-surgery orbital volume in cm$^3$; V sim = simulated post-surgery volume of orbital tissue in cm$^3$; dV = V sim - V pre; dV rel = relative difference to the largest volume of released orbital tissue in % for a particular resection scenario, i.e. lateral, floor, or two-wall (L+F) resection

## Discussion

The aim of this study was to present and computationally investigate an improved outcome of two-wall EO treatment after consideration of two special key points in lateral and floor orbital walls. Our experimental results support the hypothesis that these two key points represent natural mechanical obstacles for more efficient release of soft tissue in the extraorbital space, and have to be considered by the planning of surgical EO treatment to achieve a good result of two-wall decompression avoiding additional re-decompression.

In particular, we show that consideration of both key points enables up to 30% larger amount of decompressed orbital tissue compared to surgery scenarios ignoring these two key points. The significant effects of kp1 and kp2 can be traced back to their crucial locations: (i) resection of kp1 elevates more intraorbital tissue and provides greater mobility of the lateral rectus muscle, and (ii) resection of kp2 removes a mechanically important obstacle between the lateral and floor walls. In line with our experience, computational analysis of pre- and postoperative findings and biomechanical modelling of orbital tissue decompression can provide additional patient information and are important criteria for surgical planning. Based on individual tomographic data and consistent biomechanical modelling of soft/hard tissue behaviour, computational simulation can become an integral part of a fully digital standard for operating procedure by treatment of endocrine orbitopathy.

The main limitation of this study is the relatively small number of cases. However, it conforms with previous observations, where only one of totally two reported EO patients with unsatisfactory outcome of the first decompression treatment successfully underwent additional re-decompression [18]. Further investigations of not only two-wall, but also alternative therapeutic approaches of lateral orbital decompression (valgization) [19] are required to gain a broader evidence to reconfirm exemplary observations of this work.

## Conclusion

Our study highlights the importance of considering specific biomechanical key points for improved soft tissue outcome of EO surgical treatment. Since our computational simulations are based on parameters derived from model-fitting and postoperative data from a particular patient, the absolute values of decompression tissue volume cannot be generalized to other cases. However, because of the scalability of the model predictions, one could expect that the relative differences between simulated surgery outcomes for different resection scenarios provide a reliable estimate of the possible effects of key points. Further experimental investigations with more data are required to generalize the findings of this study.

## Methods

### Study design

The basis of the present study is the retrospective analysis of patient data diagnosed with EO in a tertiary care centre between 2010 and 2020 which have been previously described [17]. The inclusion criteria were the need for surgical rehabilitation after undergoing conservative treatment. In total, 70 patients were included in the study, of which 46 were women (65.7%) and 24 were men (34.2%). The mean age was 50.9 ± 9.8 years (range 30-76 years). 21 patients (30%) suffered from dysthyroid optic neuropathy (DON). 61 patients (87.1%) had bilateral and 9 (12.8%) had unilateral surgery, resulting in 131 orbits operated on. In most cases (72.5%), two-wall decompressions were performed. 12 patients (17.4%) underwent re-decompression surgery. In two cases a time-shifted procedure of the sides was performed. In 10 patients (14.2%, 15 orbits), the reasons were insufficient orbital decompression with less improvement of the protrusion. These patients suffered from DON in the same stage of the disease. In 7 patients (11 orbits) the re-decompression was carried further out in a new area of the medial wall (4 bilaterally and 3 unilaterally). In 4 patients (4 orbits), the re-decompression was done in the areas of our first choice of orbital decompression: 2 patients unilaterally in the region of lateral wall and 2 patients unilaterally in the region of floor wall. For our study, these four patients (mean=56.5 [SD=7.4] years, between 48 and 60 years), who underwent orbital re-decompression as a result of ineffective two-wall decompression, were examined. Accordingly, the primary variable by the assessment of surgery outcome in this study is an improved result of decompression treatment with reduction of the stigmatizing exophthalmos, symmetry of both orbits and improvement in visual acuity. The secondary variable is the improvement in double vision and orbital pressure sensation. Table 1 summarizes all important patient data. These patients suffered from EO and underwent two-wall orbital decompression (lateral and floor walls) at a major university hospital in Germany. All four patients showed insufficient results of conventional two-wall decompression which was subsequently extended to the above described two special key points leading to significantly larger release of orbital soft tissue in the extraorbital space. This led us to the idea of carrying out a biomechanical simulation of alternative scenarios of two-wall decompression.

### Image processing and geometrical modelling

Computer tomography (CT) data of a single 67-year-old male patient (patient 2) were utilized in this study for reconstruction of 3D virtual models of the patient’s orbital anatomy. The following anatomical structures were segmented using specific threshold values for each tissue. Since HU values vary within the same tissue type, they are best described as a distribution with min/max/median/mean HU values:

- bony orbit min=95, max=3071, median=893, mean=813 HU,
- intraorbital fat tissue min=-207, max=-36, median=-93, mean=-92 HU,
- orbital muscles min=-50, max=86, median=1, mean=0 HU,
- eyeball (bulbus) min=-10, max=81, median=32, mean=32 HU.

Based on segmented CT images, 3D unstructured triangulated surfaces and tetrahedral volumetric meshes of the above tissues were generated using the Amira 4.1 Visualization and Geometrical Modelling Package (Mercury Computer Systems, Chelmsford, MA, USA). Subsequently, pre- and post-surgery models were registered using a rigid transformation based on a set of manually defined bony skull landmarks. The resected lateral and floor orbital walls were labelled for subsequent computational simulation of soft tissue decompression that consisted of predicting orbital fat and muscle tissue displacement in response to resection of orbital walls.

### Biomechanical modelling of decompression surgery

Simulation of soft tissue relocation for different scenarios of two-wall resection was performed using the finite element method (FEM). For a detailed description of the FEM model used in this study, we refer to our previous works [20]. The material model used in the present simulation relies on an isotropic, homogeneous, and piece-wise linear elastic approximation of mechanical soft tissue behaviour described by the linearized stress–strain relationship:

1 $$\begin{aligned} \sigma (\varepsilon )~=~\frac{E}{1+\nu }\,\left( \varepsilon +\frac{\nu }{1-2\nu }tr({\varepsilon }){\mathbf {I}} \right) \,, \end{aligned}$$

where $\sigma$ denotes the Cauchy stress tensor (i.e. external forces), $\varepsilon$ is the Green–Lagrange strain tensor (i.e. material deformation), ${\mathbf {I}}$ is an identity matrix, and ($E, \nu$) are two basic material parameters (the modulus of elasticity and the Poisson’s ratio, respectively) describing the stiffness and compressibility of the Hook’s material. Since boundary value problems in surgery planning are given in a pure geometrical form (i.e. images and not real material, displacements and not forces), the material parameters in Eq. 1 are not known. Consequently, the modulus of elasticity and the Poisson’s ratio are often estimated from exemplary measurements of tissue properties in cadavers or animals [21]. However, soft tissue properties can vary substantially and adaption of some literature values completely disregards individual tissue behaviour. In this work, the missing material parameters as well as the effective forces were estimated using a direct comparison with postoperative data. In particular, the following parameters were iteratively estimated from a comparison of repetitive model predictions vs. post-surgery outcomes of two-wall orbital resection: (i) relative stiffness of muscle to soft tissue; (ii) compressibility of soft tissue, and the effective forces applied to (iii) lateral, and (iv) bottom of orbital walls. Thereby, the force field driving the outflow of soft tissue from the orbital space was naturally assumed to act along the normal to the orbital surface. The introduction of different forces at the lateral and bottom walls is necessary because of essentially different boundary conditions such as the presence of a muscle layer in the case of later walls, and almost no resistance to released soft tissue on the orbital bottom. The four-dimensional parameter space was sampled by totally 320 equidistant sampling points (i.e. 320 combinations of four parameter values). The optimal set of parameters was sought as the global minimum of the difference between the simulated and real post-surgery volume soft tissue released in the extraorbital space. The set of parameters with the minimum difference between simulated and real post-surgery decompression was used for subsequent simulation of different wall resection scenarios.

## Data Availability

The datasets used and/or analysed during the current study are available from the corresponding author on reasonable request.
